# My Wealth, (Y)Our Life Satisfaction? Sole and Joint Wealth Ownership and Life Satisfaction in Marriage

**DOI:** 10.1007/s10680-022-09630-7

**Published:** 2022-08-30

**Authors:** Nicole Kapelle, Theresa Nutz, Daria Tisch, Manuel Schechtl, Philipp M. Lersch, Emanuela Struffolino

**Affiliations:** 1 Department of Sociology, Nuffield College, The Leverhulme Centre for Demographic Science, University of Oxford, 42-43 Park End Street, Oxford, OX1 1JD UK; 2 GESIS-Leibniz Institute for the Social Sciences, Mannheim, Germany; 3 Humboldt-Universität Zu Berlin, Berlin, Germany; 4 Max Planck Institute for the Study of Societies, Cologne, Germany; 5 DIW Berlin, Berlin, Germany; 6 University of Milan, Milan, Italy

**Keywords:** Subjective well-being, Wealth, Marriage, Individualisation, Family economics, Gender

## Abstract

This study examines the money-subjective well-being nexus by studying the link between changes in jointly and solely (i.e. respondents’ own and their partner’s own) held gross wealth and changes in married individuals’ subjective well-being. Joint assets reflect norms of sharing responsibilities and resources. Solely held assets, in contrast, offer individual economic independence. Using wealth data from the German Socio-Economic Panel Study (SOEP; 2002, 2007, 2012, 2017), we estimate individual fixed effects regressions. Although coefficients for all three wealth measures are positive, our results highlight that only increases in jointly held wealth are associated with statistically significant increases in spouses’ life satisfaction in Germany. Despite expectations about a stronger relevance of joint wealth for men compared to women in line with men’s role as a financial provider for the family, we do not find substantial gender differences in the positive association between increases in joint wealth and life satisfaction. In light of the individualisation of marriages, our results highlight that the personal benefits associated with marital sharing of wealth seem to trump those of economic independence and financial autonomy.

**Supplementary Information:**

The online version contains supplementary material available at 10.1007/s10680-022-09630-7.

## Introduction

In the continuation of the “Second Demographic Transition” (Van De Kaa, 1987), the meaning of marriage has shifted from companionate towards more individualised partnerships, where partners’ personal choices and self-fulfilment have become increasingly important (Cherlin, 2004). With the individualisation of marriage, individual compared with unitary preferences of both partners, emotional companionship, and personal autonomy have gained importance. At the same time, separation and divorce have become common features of family life courses. In light of these factors, stronger preferences for keeping money separate and a reduced need for economic support within marriage are expected—particularly within newly formed marriages (Yodanis & Lauer, 2014). However, the social norm of marital sharing remains strong (Pepin, 2019; Tisch & Lersch, 2021), and many couples continue opting for (at least partial) resource pooling (Yodanis & Lauer, 2014). In addition, marriage plays a vital role in facilitating the accumulation of economic resources, for instance, through tax benefits and joint savings (Kapelle & Lersch, 2020). Amid this (rising) economic complexity within couples, the consequences of wealth ownership within couples are under-researched.

In the present study, we examine how changes in solely held wealth (i.e. wealth held only in one partner’s name) compared with changes in jointly held wealth (i.e. wealth held in both partners’ names) are related to changes in individuals’ subjective well-being (i.e. individuals’ overall evaluations of their satisfaction with life) within marriage. Solely owned wealth provides financial autonomy and independence, while jointly owned wealth adheres to norms of marital sharing and spreads financial obligations of ownership across two pairs of shoulders. We focus on different-sex, first-time married couples aged 18 and older^1^ because of (historically) different legal rights between different-sex, same-sex and cohabiting couples in many contexts including Germany—the country case of the current study. Higher-order marriages are also characterised by substantially different monetary behaviour compared to first marriages (Burgoyne & Morison, 1997; Kan & Laurie, 2014).

Wealth is distinct from income as a measure of material prosperity. Beyond potentially volatile, current income flows, wealth provides long-term economic security (Killewald et al., 2017; Spilerman, 2000). Wealth acts as a safety net protecting against future hardship (e.g. unemployment, retirement), guarantees long-term consumption potential, confers power and status, can be transferred to successive generations, and secures direct benefits by its use-value (e.g. ownership of a home) (Keister, 2000). Whereas income is mostly individually earned irrespective of the marital status, wealth in marriage can be the product of individual and joint savings, investments, and transfers. In most matrimonial property regimes, including the default regime in Germany, spouses maintain individual property rights during marriage (Nutz, Nelles, & Lersch, 2022). Thus, wealth owners can make largely independent decisions on their personal wealth, which potentially affects their well-being and makes it necessary to differentiate the ownership status within couples. See Frémeaux and Leturcq (2022) in this Special Issue for a discussion of the French property regime and its link to between- and within-household differences in wealth accumulation.

Research has illustrated that wealth is positively associated with subjective well-being in affluent societies (Brulé & Suter, 2019; D’Ambrosio et al., 2020; Headey et al., 2008). Following the widespread notion of marital sharing and perceptions of the marital household as a unit (Becker, 1993), these previous studies on the nexus between wealth and subjective well-being refer to the household as the unit of analysis. Thus, potential within-couple inequalities in wealth and subjective well-being have been largely ignored.

Challenging this questionable assumption of a fully unitary household and emphasising individualisation tendencies (Bennett, 2013), an incipient body of recent literature started to illustrate the relevance of couples’ wealth arrangements for spouses’ subjective well-being (Kan & Laurie, 2014; Lersch, 2017; Tisch, 2021). While these studies provide critical insights into the black box of the family, several shortcomings are noteworthy. First, all studies only capture couples’ wealth ownership structure to a limited extent. While Kan and Laurie (2014) use two dummy indicators for the presence of any joint or sole wealth, Lersch (2017) and Tisch (2021) use levels of own and partner’s personal wealth which reflects the sum of jointly and solely held wealth. Thus, none of the studies explicitly disaggregates couples’ wealth into each partner’s solely held wealth and their jointly held wealth to fully reflect couples’ wealth ownership. Second, due to data limitations, only Tisch (2021) fully leverages the panel structure of the analysed data, while Kan and Laurie (2014) and Lersch (2017) had to rely on cross-sectional methods using pooled multi-year data.

We address these shortcomings in the present study by using detailed, longitudinal wealth information from the German Socio-Economic Panel Study (SOEP; 2002, 2007, 2012, 2017) that allows us to fully disaggregate couples’ wealth into each partner’s sole and their joint wealth. We thus fully account for the wealth ownership structure of couples and go beyond more simplistic measures of wealth ownership in prior research. This enables us to critically evaluate expectations from competing theoretical approaches—including the unitary household model, gendered norms, and individualisation. By using longitudinal data to estimate fixed-effects regression models, we provide more robust evidence than was previously possible (see Tisch (2021) for an exception). Knowing more about the consequences of financial arrangements in couples is conducive to a better understanding of how these arrangements could indirectly affect demographic outcomes linked to subjective well-being, such as health, longevity, and parenthood (Cetre et al., 2016; Diener & Chan, 2011), or shape how demographic processes related to the individualisation of marriage may affect subjective well-being within couples. In sum, we significantly expand previous research and make substantial contributions to the literature on marriage, wealth, and subjective well-being.

## Background

### Defining Subjective Well-Being

Subjective well-being is a meaningful measure of social welfare and an indicator of economic and social progress (Hochman & Skopek, 2013; Jantsch & Veenhoven, 2019). Subjective well-being is a multidimensional concept that covers multiple areas of life, including dimensions of emotional, health-related, and social well-being. In this study, we focus on individuals’ overall self-assessed evaluations of their life—that is, their well-being subjectively evaluated or their life satisfaction. Life satisfaction is one of the most commonly used concepts for individuals to assess their subjective well-being (Abdallah & Mahony, 2012; Hochman & Skopek, 2013; Keizer & Komter, 2015). We, therefore, use the terms life satisfaction and subjective well-being interchangeably.

### The Nexus Between Wealth and Subjective Well-Being

Three theoretical approaches explain the relationship between financial resources and subjective well-being. First, according to the *needs hypothesis*, money increases subjective well-being by helping individuals meet their objective or subjective needs, such as food and shelter, financial security, social status, education, or self-fulfilment (Diener & Biswas-Diener, 2002). Wealth specifically might positively affect individuals’ life satisfaction by supporting them in meeting their needs and increasing their personal freedom, feelings of financial security, and scope of influence or power. Importantly, wealth may act as a buffer against certain life events and shocks (e.g. unemployment and ill-health) (Brulé & Suter, 2019; Kuhn & Brulé, 2019).

Second, the *relative standards model* and *social comparison theory* propose that individuals use various reference points to evaluate their well-being (Easterlin, 2001). These reference points could be located in individuals’ past or future (e.g. own past financial resources or future financial goals). However, they could also emerge from the social context (e.g. in comparison with significant others’ financial standing). Therefore, wealth may increase individuals’ subjective well-being whenever the current financial standing is perceived to be better than own financial standing of the past or the financial standing of others.

Last, the *cultural approach* suggests that individuals evaluate their well-being based on cultural scripts about financial resources. Culture shapes individuals’ goals regarding wealth accumulation (Diener et al., 1999). Accordingly, if individuals’ behaviour is consistent with cultural norms about wealth accumulation, they are expected to gain subjective well-being because they experience positive emotions socialised to these norms (Diener & Biswas-Diener, 2002). Within Germany, the country context of the present study, strong social norms about expectations to save, be frugal, and accumulate wealth are prevalent. This is, for instance, reflected in widespread capital investments in less risky assets (e.g. saving deposits for private households in Germany). In contrast, investment rates in risk-prone investments and credit acquisition are higher in other countries (Borsch-Supan, 2003).

Based on these three theoretical approaches, wealth levels and changes in these levels are expected to play a significant role in subjective well-being. Nevertheless, empirical research has started to examine this relationship only recently (e.g. Brulé & Suter, 2019; Hochman & Skopek, 2013). The vast majority of prior empirical studies examined the money-subjective well-being nexus with a focus on income rather than wealth (for a review on the income literature, see Diener and Biswas-Diener (2002) or Tay, Zyphur, and Batz (2018). Yet, the incipient research that considers wealth has highlighted that wealth may be more important for subjective well-being than income (e.g. Brulé & Suter, 2019; Headey et al., 2008).

However, in most cases, these prior studies refer to the household as their unit of analysis and thus assume that household members equally benefit from wealth increases. This focus on the household as a single unit in line with Becker’s (1993) unitary household model is problematic from the perspective of the current study. A growing body of research has highlighted that although family life provides a range of economic benefits, not all economic resources are fully pooled and shared within households (e.g. Bennett, 2013; Çineli, 2022; Joseph & Rowlingson, 2012; Nutz & Gritti, 2021). Although spouses and their economic and subjective well-being are inevitably linked, individuals also act independently from their spouses. Thus, for the focus on married spouses, previously introduced theoretical notions have to be considered in light of couple and individual dynamics (Blood & Wolfe, 1960).

### Previous Research on Wealth within Couples and Spouses’ Subjective Well-Being

To the best of our knowledge, only three studies are opening the black box of couples’ wealth and spouses’ subjective well-being (Kan & Laurie, 2014; Lersch, 2017; Tisch, 2021). By pooling different waves of the British Household Panel Study, Kan and Laurie (2014) examine the association between the ownership of wealth and subjective well-being, measured as respondents’ self-reported psychological well-being. Wealth ownership is operationalised by binary indicators measuring whether different wealth components—specifically savings, investments, or debt—are available and held jointly or solely. However, it can be expected that most couples hold at least some wealth. Thus, the approach by Kan and Laurie (2014) uses a potentially selective group as their reference (i.e. couples without joint or sole wealth). Furthermore, as the authors do not include an indicator for whether respondents’ partners hold any sole wealth, the study only partially accounts for the wealth ownership structure within couples. Relevant to the current study, Kan and Laurie (2014) show that British women’s and men’s psychological well-being is positively associated with the (binary) ownership of solely and jointly owned savings. Gender differences emerge for their investment indicators. While any ownership of investments, whether it is jointly or solely held, is positively associated with women’s well-being, only men’s sole ownership of investments is positively associated with their well-being. Although this study provides a first indication on the link between wealth ownership structure and spouses’ subjective well-being, it leaves key questions unanswered with regard to (a) the extent to which the amounts of jointly and solely owned wealth are relevant, (b) the relationship between changes in ownership and well-being, and (c) the role of spouses’ partner’s sole ownership.

Lersch (2017) focuses on the level of wealth and pools several waves of the German Socio-Economic Panel to examine how respondents’ personal and their spouses’ wealth are related to subjective financial well-being. He defines personal wealth as the sum of solely held wealth and the individual share of jointly held wealth, thus disregarding the ownership structure. Lersch finds that married women’s financial well-being is equally associated with their individual wealth levels and their spouses’ wealth in older birth cohorts. In birth cohorts born after 1965, women’s financial well-being is more strongly associated with their individual wealth than their spouses’ wealth. For men, individual wealth and spouses’ wealth is positively related to men’s financial well-being, with the association being larger for individual wealth. This study, however, did not distinguish between solely and jointly owned wealth.

Tisch (2021) studies changes in respondents’ own personal, their partners’, and overall couple’s total gross wealth using longitudinal data from the German Socio-Economic Panel. She shows that increases in any of the three gross wealth measures are positively associated with life satisfaction for women and men. However, similar to Lersch (2017), this study does not differentiate between sole and joint wealth, making it unfeasible to draw more in-depth conclusions on how changes in solely and jointly owned wealth are related to life satisfaction.

In this study, we build on these three seminal studies and estimate the association between each partner’s solely held wealth and couples’ jointly held wealth and spouses’ life satisfaction. By distinguishing solely held (i.e. two continuous measures of each partner’s solely held resources) and jointly held wealth (i.e. one continuous measure of the couple’s jointly held resources), we study the relevance of wealth levels rather than couples’ wealth management. We compare how changes in the three different components of couples’ wealth are associated with changes in spouses’ life satisfaction using longitudinal data and methods that provide more robust evidence of a causal effect.

### Couples’ Wealth Ownership and Individuals’ Subjective Well-Being

In the following section, we integrate the couple perspective into the theory on the nexus between wealth and subjective well-being to derive our hypotheses. We explicitly consider that depending on how wealth is held (i.e. solely compared to jointly) and in which partner’s hands (i.e. spouse’s sole or spouse’s partner’s sole wealth), spouses could experience different benefits or disadvantages that may be linked to their subjective well-being.

Overall, both jointly and solely held wealth can provide essential economic resources to cover the needs of the household and its members. This found support in previous research of Kan and Laurie (2014), Tisch (2021), and Lersch (2017), who showed that holding any wealth or increases in any wealth are generally positively associated with subjective well-being (though not differentiating levels in sole and joint wealth). In line with this evidence, we derive a general, first hypothesis:

#### H1 Wealth hypothesis

*Increases in solely and jointly held wealth and partner’s solely held wealth are positively related to increases in respondent’s subjective well-being.*

However, we may expect relevant differences in how well-being increases based on how wealth is held. In order to fulfil individual needs using joint wealth, it should be assumed that spouses have sufficient access to joint wealth in line with Becker’s (1993) unitary household model. However, as previously mentioned, prior research shows that husbands and wives do not always have equal access to and control over joint financial resources (Bennett, 2013; Joseph & Rowlingson, 2012) and spouses might have different preferences about how to spend or invest financial resources (Lundberg et al., 1997). Thus, access to joint wealth may need to be negotiated. On the contrary, solely held wealth has the advantage that owners can—within certain limits—freely decide how to manage and whether or when to consume, invest, or save their wealth. Thus, personal property rights of solely held wealth provide an individual safety net and a buffer against economic hardship that does not require to be negotiated with the spouse (Brulé & Suter, 2019).

Furthermore, spouses might see each other as the respective reference point as suggested by the relative standards model. In that case, individuals’ life satisfaction might increase if their solely held wealth increases in comparison with their partner’s solely held or jointly held wealth (Tisch, 2021). This is in line with the resource theory of power (Blood & Wolfe, 1960), arguing that relative wealth within couples determines the bargaining power in financial and non-financial decisions. Thus, increases in solely held wealth may strengthen the individual’s stance in negotiating compromises concerning investments and purchases of the household, which might again increase life satisfaction.

According to the cultural approach, joint wealth may affect subjective well-being positively because couples that hold wealth jointly fulfil the prevailing cultural script of marital sharing. Accumulating wealth jointly is explicitly part of the “marital script”, a set of normative roles and responsibility expectations for married men and women (Dew, 2016). This script emphasises joint investments for the family’s future (e.g. children and retirement). It thereby provides a sense of long-term mutual commitment to the partnership and the possibility to rely on the partner financially. Despite growing individualisation, marriage remains a public commitment to a shared, long-term future together (Holland, 2013; Poortman & Mills, 2012; Tisch & Lersch, 2021). Pooling at least some financial resources also remains the norm in most married couples (Yodanis & Lauer, 2014). In contrast, increases in solely held wealth violate the marital norm of sharing, which may be socially sanctioned. In addition, partners may perceive increases in solely held wealth as a signal of mistrust and weak partnership commitment, reducing life satisfaction.

Moreover, increases in joint wealth might offer the possibility to fulfil individual needs which could not be fulfilled with solely held wealth alone. For example, homeownership—at least in Germany—is mainly conditional on spouses’ joint investment because of Germany’s prudential mortgage system and large down payments (Voigtländer, 2009). Furthermore, joint wealth has the advantage that financial risks are pooled. For instance, one partner’s temporary inability to pay for their mortgage share may be compensated by the other partner. Thus, individuals’ vulnerability in the case of expected and unexpected adverse events is reduced, which would otherwise affect an individual’s financial situation negatively (e.g. unemployment and poor health).

Thus, while increases in respondents’ sole wealth, their partner’s sole wealth, and joint wealth may provide some advantages and disadvantages, we argue that the cultural script of marital sharing is particularly evident in the German institutional case. Germany is often portrayed as a conservative, familistic, and traditional welfare regime that encourages or favours traditional gendered division of labour within the family. Married couples in Germany are confronted with a broad set of policies that treat the household as a single unit. Married spouses and parents can, for instance, profit financially from joint tax filing, tax reductions, or joint insurances (e.g. health care insurance) and pensions, particularly if spouses’ earnings are unequal (Bach et al., 2013; Buslei & Wrohlich, 2014). Such marital advantages rest on traditional ideas about gender roles and specialisation within marriage (Lundberg & Pollak, 1993; Parsons, 1949). Germany’s strong institutional incentives for married couples to think jointly and for spouses to adopt a traditional division of labour are also reflected in comparatively gendered social norms (Aisenbrey & Fasang, 2017; Trappe et al., 2015). As a result, German women earn less labour market income and own less wealth than their husbands, often implying the lack of substantial independent resources for women (Grabka et al., 2015; Nutz & Gritti, 2021; Trappe & Sørensen, 2006).

In light of the traditional institutional setting that encourages jointness, we expect couples to adhere to the cultural script of marital sharing and joint thinking within the context under study. Thus, we derive the following:

#### H2 Joint wealth hypothesis

*Increases in jointly held wealth are more strongly positively related to increases in subjective well-being than increases in respondents’ solely held wealth or partner’s solely held wealth.*

Moreover, we may foresee own sole and partner’s sole wealth to be differently associated with life satisfaction. Although solely held wealth benefits the owner, the owner’s partners might also benefit from these resources within the partnership. For example, if non-financial assets such as owner-occupied housing are solely held, the owner’s partner may nevertheless benefit from the accommodation of this asset, affecting their subjective well-being positively. In addition, in the German context, spouses are legally obliged to share some of their wealth under certain conditions—such as unemployment. However, inconsistently with the norm of marital sharing, individuals have no legal rights to participate in their spouse’s wealth according to Germany’s default marital property regime of the community of accrued gains (Kapelle & Baxter, 2021). Consequently, individuals may not feel entitled to use their partners’ solely held wealth even if they have the partner’s permission. Additionally, access to the partner’s wealth would also need to be negotiated. We, therefore, expect that:

#### H3 Sole wealth hypothesis

*Increases in solely held wealth are more strongly positively related to increases in *subjective* well-being than increases in partner’s solely held wealth.*

In contexts where traditional gender ideologies are prevalent—such as Germany—men are seen as the household’s primary financial provider and expected to adhere to gendered norms to provide sufficient resources for dependent family members. Women, in contrast, are seen as primary carers and responsible for the majority of unpaid labour in the household. The traditional division of labour might lead partners to expect men to provide jointly owned assets (Pepin, 2019). Although women might also gain life satisfaction through their male partner’s breadwinning, especially men who actively fulfil their role as the financial provider via investments in joint wealth are expected to benefit from increases in joint ownership in terms of life satisfaction. That is, men gain in subjective well-being more than women do if they adhere to the social norms of sharing wealth when joint wealth increases net of their solely owned wealth. About these gender differences, we expect:

#### Hypothesis 4 Family provider hypothesis

*Increases in jointly held wealth are more strongly positively related to increases in subjective *well*-being for men than for women.*

## Data and Methods

### Data

We use high-quality longitudinal data from the German Socio-Economic Panel Study (SOEP, v36; Goebel et al. (2019)), a representative survey of German households that commenced in 1984. The data are well-suited for the present study as they provide (a) longitudinal information on respondents’ life satisfaction, (b) detailed information on retrospective marital biographies updated yearly with prospective data, and (c) comprehensive measures of wealth within couples. While life satisfaction is measured every year, wealth data are collected in 2002, 2007, 2012, and 2017. The wealth module covers questions on many asset and liability components, including information on financial assets, primary housing values, or outstanding mortgages and credit card debts.

The SOEP is unique in the way that it collects wealth data exclusively at the individual level following a stepwise procedure: First, a filter question (yes/no) is asked to assess whether the respondent personally holds a specific type of asset or liability. Second, in case of an affirmative answer, respondents are asked to provide the total market value of the asset or liability. Third, for wealth components that can theoretically be owned jointly (e.g. housing equity), a second filter question (yes/no) is asked to assess whether the asset or liability is held jointly. Fourth, if respondents affirm joint ownership, they are asked to provide their personal share in percentage points. For a detailed description of the wealth data, including a list of wealth components and data collection strategies, see Table A1 in Online Appendix.

For our analyses, we use wealth data that were edited and multiple imputed by the SOEP survey team (see Grabka and Westermeier (2015) for detailed descriptions of the process). Building on these imputations, we additionally impute missing data with chained equations for all analytical variables and a range of auxiliary variables using Stata’s *mi* procedure (version 16.1). A total of *m* = 10 imputed data sets were created (see Online Appendix, Table A2 for an overview of missing values). Estimation results from ten imputed data sets are combined using Rubin’s rule (Rubin, 1987).

### Sample

We restrict the analytical sample to currently married different-sex couples in which both partners are married for the first time, are at least 18 years of age, and provided valid interview responses. Thus, for first-time married couples that experience the dissolution of their marriage (i.e. separation, divorce, widowhood) during the observational window (i.e. 2002 to 2017), we drop all person-years observed after the marriage ends. Further, we exclude couple-years in which married couples lived with other adults (e.g. multi-generational households, house or flat shares, etc.), leading to the exclusion of 375 couple-years (1.70 percent of all couple-years) and total exclusion of 173 couples (1.47 percent of couples). Additionally, we drop 536 couple-years (2.47 percent of all couple years) in which at least one partner did not provide any information on wealth, which results in the total exclusion of 250 couples (2.16 percent of all couples). This restriction is mainly necessary because wealth data were not collected for all respondents in the refreshment samples in their first observation. Thus, the total exclusion of couples in this step of the sample restriction results from a lack of repeated observations.

Finally, we restrict the sample to couples observed in at least two waves because we use a fixed-effects regression approach that relies on within-unit variation. The final analytical sample consists of 5,866 couples (11,732 individuals) with 15,685 couple-year observations (31,370 individual-year observations). The sample is unbalanced, with 56 percent of couples observed in at least two waves, 21 percent observed in three waves, and 23 percent observed in all four waves. In the analyses, each individual in the sample of married couples is considered twice: once as an anchor person and once as the partner of the anchor.

### Measurement

#### Outcome Variable

Our outcome variable is subjective, self-assessed well-being, measured as overall life satisfaction. In the SOEP, respondents are asked to assess “[…] (their) satisfaction with (their) life in general” in a single item on an 11-item scale ranging from “completely dissatisfied” to “completely satisfied” (see Fig. A1 in Online Appendix for a graphical illustration of the distribution). Although self-assessed well-being does not equally represent each underlying dimension of well-being, such as psychological or mental health or trust in the community, it is found to be broadly representative of individuals’ *overall* subjective well-being (Jeffrey, Abdallah, & Quick, 2015).

#### Main Explanatory Variables

The choice of the explanatory variables follows the theoretical discussion in the previous sections. To measure wealth ownership structures within couples, we include three main explanatory variables measuring levels of log-transformed gross wealth that (1) respondents’ hold solely, (2) respondents’ partners hold solely, and (3) respondents hold jointly with their partners. We consider gross wealth (i.e. the sum of assets excluding debts and liabilities) because net wealth calculated as the difference between gross wealth and debts potentially masks existing ownership structures within couples. Additionally, debt is likely differently associated with life satisfaction than gross wealth. Depending on the type of debt (e.g. mortgage, outstanding credit card payments, student debt), it may entail relevant responsibilities or may be a sign of financial difficulty, which in return may be negatively associated with life satisfaction. While detailed explorations of the association between spouses’ debt and their life satisfaction are beyond the scope of the current study, we consider debts by adjusting for respondents’ and their partners’ log-transformed personal debt and liability levels (i.e. the sum of all personally owned debts and liabilities and the personal share of any jointly held debts and liabilities). All wealth measures are adjusted for inflation and top-coded at the 0.1 percent level. Additionally, we add one Euro to each raw wealth measure prior to the log-transformation to avoid the exclusion of respondents with no wealth. To address whether effects differ by gender, we additionally generate a dummy variable for gender (male [ref.], female).

#### Covariates

In addition to the previously mentioned debt measures, we account for other relevant time-varying covariates.^2^ Observed and unobserved time-constant covariates are automatically captured by our fixed-effects regression approach and hence not included. Specifically, we account for both partners’ ages using a categorical variable (aged 39 or younger [ref.], 40 to 49, 50 to 59, 60 to 69, and aged 70 and older) to consider maturation effects and a potentially u-shaped development of life satisfaction over the life course. In addition, to account for changes in life satisfaction throughout the marriage, we adjust for the duration of the marriage using a categorical variable (married for up to 10 years [ref.], 10 to 19 years, 20 to 29 years, and married for 30 or more years).

Additionally, we account for a range of economic and human capital factors, including respondents’ and their partners’ employment level (full-time [ref.], part-time/irregular, not employed/other), self-employment (yes [ref.], no), and income (personal log-transformed gross annual labour income). We further adjust the models for the household’s region (urban [ref.], rural) and the area of living (Eastern Germany [ref.], Western Germany). Finally, we also account for the household’s receipt of inheritances within the last five years to capture windfall gains. Note that for 2002, the dummy only indicates the receipt of household-level inheritance in 2000, 2001, and 2002 as a comprehensive inheritance measure was only introduced into the SOEP in 2000.^3^

We decided against adding other family-related variables favouring a more parsimonious model. We also want to avoid bias due to over-controlling. For robustness checks, we added three continuous variables to capture the presence, age, and number of dependent children living in the household (number of children aged 0 to 4 years, number of children aged 5 to 10, and number of children aged 11 to 18) as well as two continuous measures of the number of children respondents’ and their partners’ have ever had to our regression models, which did not change our main results (see Figure A2 in Online Appendix).

### Analytical Strategy

After presenting descriptive evidence, we deploy fixed-effects regressions to leverage the panel data and model the relationship between wealth ownership and life satisfaction. We begin with the following model for repeated observations nested within individuals:

$${\text{y}}_{it}= \mu +{\varvec{\beta}}{\text{W}}_{it}+{{\varvec{\delta}}{\text{X}}}_{it}+{\varvec{\gamma}}{\text{Z}}_{i} + {\alpha }_{i}+ {\varepsilon }_{it}$$

where subscript $i$ denotes individuals and subscript $t$ denotes time period. We denote our outcome variable life satisfaction $y$ which varies between and within individuals over time. ${\text{W}}_{it}$ denotes our continuous measures of the anchor person’s sole wealth, their partner’s sole wealth, and joint wealth. ${\text{X}}_{it}$ is a vector of time-varying covariates, while ${\text{Z}}_{i}$ is a vector of time-constant covariates. $\mu$ is the intercept and ${\varvec{\beta}}$, ${\varvec{\delta}}$ and ${\varvec{\gamma}}$ are vectors of coefficients. Other than in cross-sectional regression, longitudinal data allow the error term to be split into ${\alpha }_{i}$ and ${\varepsilon }_{it}$. While ${\varepsilon }_{it}$ denotes the stochastic error term that varies across individuals and over time, ${\alpha }_{i}$ denotes the combined effect of time-invariant, individual-specific heterogeneity and hence only differs across individuals but not over time.

We estimate individual fixed-effects models by mean-differencing of outcome and explanatory variables for each respondent across all available time points. This means that we compare the same individual over time using at least two time points. Time-invariant terms, ${\text{Z}}_{i}$ and ${\alpha }_{i},$ are hence averaged out. Our fixed-effects models can therefore produce estimates of $w$ on $y$ that implicitly account for any observable and unobservable time-constant heterogeneity. Overall, our results show how a one-unit change in log-transformed gross wealth relates to changes in life satisfaction.

To address our four hypotheses, we run a set of fixed-effects regression models. To test our first three hypotheses, we predict life satisfaction using our three outcome measures and additionally assess and test whether differences between coefficients are substantially and statistically significant. In a second step, we examine our remaining hypothesis on gender differences by interacting our three wealth measures with our binary gender variable.

## Results

### Descriptive Results

Table 1 shows descriptive statistics of the sample overall and disaggregated by gender (see Table A3 in Appendix for a more detailed description of the sample). Overall, individuals hold more joint wealth than sole wealth. With around 94,000 Euros, the value of joint wealth is almost double the value of individuals’ or their partners’ sole wealth (53,000 Euros, respectively). However, in line with research on within-couple wealth inequalities (Kapelle & Lersch, 2020), sole wealth levels differ substantially once we disaggregate the sample description by gender. Women hold on average 31,000 Euros solely while men own 75,000 Euros solely. In our sample of married respondents, 61 percent of respondents own a home. In addition, 91 percent of homeowners own their homes jointly with their spouses.

**Table 1:** Descriptive statistics of outcome and explanatory variables

	Total		Women		Men	
	Mean/Prop	SD	Mean/Prop	SD	Mean/Prop	SD
*Outcome measure:*						
Life satisfaction	8.25	1.61	8.28	1.61	8.22	1.61
*Gross wealth:*						
Sole gross wealth (EUR)*	52.97	276.99	30.73	115.98	75.21	372.84
Sole gross wealth partner (EUR)*	53.12	279.84	75.56	376.37	30.67	118.18
Joint gross wealth*	93.86	164.67	92.83	155.92	94.90	172.98
Personal debts and liabilities*	23.72	67.67	21.16	49.28	26.28	81.95
Partner debts and liabilities*	23.78	68.00	25.76	69.75	21.79	66.14
Homeownership (ref. no homeownership)	0.61		0.61		0.61	
Sole homeownership (ref. joint homeownership)	0.09		0.06		0.12	

*Gross wealth and debts reported in 1,000 EUR. Slight deviations in the distribution of anchor’s and partner’s solely owned gross wealth result from variation generated through the multiple imputation procedure at the individual level.

*Data:* SOEP (v36); unweighted, multiply imputed

**Fig. 1:**
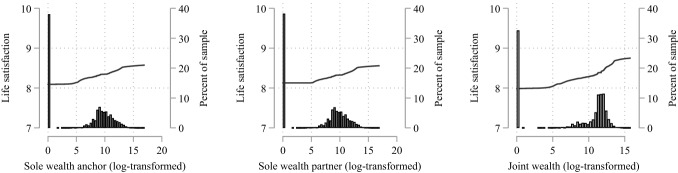
Bivariate associations between sole and joint gross wealth and life satisfaction *Notes*: Locally weighted running-mean smooth and histogram. Illustration based on the first imputation set. *Data*: SOEP (v36); unweighted, multiply imputed

Figure 1 pools data for our four wealth waves and shows the bivariate association between sole and joint gross wealth and life satisfaction for individuals in our analytical samples, using a locally weighted running-mean smooth (left y-axis). Although our life satisfaction variable ranges from 1 to 11, we only graph the range of 7 to 10 to better illustrate how life satisfaction varies across the wealth distribution in the range where most respondents locate their life satisfaction. The distribution of wealth across the sample is shown in a histogram (right y-axis). As shown in Fig. 1, individuals’ life satisfaction is lowest if either they or their partners do not own sole respectively joint wealth and increases with the amount of wealth held. The difference in life satisfaction between individuals at the bottom of the wealth distribution and those at the top of the distribution is largest for joint wealth, suggesting that the amount of jointly held wealth is significant for individuals’ life satisfaction.

### Regression Results

**Fig. 2:**
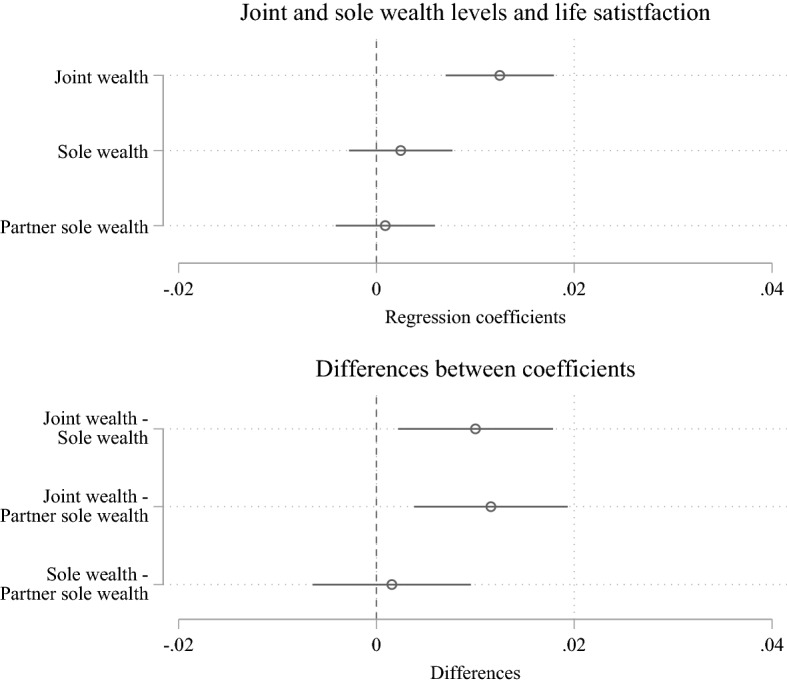
Multivariable fixed-effects regression models of sole and joint gross wealth (log-transformed) on life satisfaction *Notes:* Whiskers indicate 95% confidence intervals. The models are also adjusted for both partners’ personal liabilities, age, marital duration, employment level, self-employment, and income. Additionally, we account for the household’s region with regard to rural compared to urban and Western compared to Eastern Germany, and the receipt of inheritances. Full model results in Online Appendix Table A4. *Data:* SOEP (v36); multiply imputed

Figure 2 depicts the results of multivariable fixed-effects regression models of respondents’ own and their partners’ sole gross wealth and joint gross wealth on life satisfaction. Whereas the upper panel shows the regression coefficients, the lower panel plots the differences between the coefficients to test hypotheses that compare sole and joint wealth.

In our *Wealth Hypothesis* (H1), we expected that increases in any of the three gross wealth variables—own sole wealth, partner’s sole wealth, or joint wealth—would be associated with increases in married respondents’ life satisfaction. As shown in the upper panel of Fig. 2, effects sizes for all three gross wealth coefficients are positive. Specifically, a one-unit increase in log-transformed joint wealth is associated with a 0.012 increase in life satisfaction, statistically significant at the 5 percent level. In other words, our results indicate that a doubling of joint wealth is associated with an increase of approximately 0.01 life satisfaction points where life satisfaction has a within-individual standard deviation of 0.9. In contrast, increases in respondents’ and their partner’s log-transformed sole wealth by one unit are only associated with 0.002 and 0.001 unit increases in life satisfaction, respectively, which are statistically insignificant effects. Although the effect size for joint wealth is the largest among all other wealth measures included in our analyses, it is arguable if the effect size of joint wealth is substantial. We argue that it has to be acknowledged that our analyses additionally account for income. Thus, we show that wealth and specifically joint wealth is relevant even beyond income. Furthermore, we focus on within-individual changes with our fixed-effects analyses, which likely renders smaller effect sizes compared to cross-sectional analyses. Finally, prior literature on the money-subjective well-being nexus found similarly small effect sizes (Headey et al., 2008; Tisch, 2021).

For an added perspective, we look at the effect size of some other control variables. A transition from our reference age bracket (i.e. respondents aged 39 and younger) to the second age bracket (i.e. respondents aged 40 to 49) is associated with a statistically significant 0.080 decrease in life satisfaction. A relocation from Western to Eastern Germany is associated with a life satisfaction decline of 0.384 and the experience of unemployment compared to full-time employment is linked to a 0.171 reduction in life satisfaction.

As already visible based on differences in the effect sizes of our three gross wealth measures, our results indicate substantial variation in the extent to which our three wealth measures are associated with life satisfaction. This was anticipated within our *Joint Wealth Hypothesis* and *Sole Wealth Hypothesis*. Specifically, we hypothesised in the *Joint Wealth Hypothesis* (H2) that increases in joint wealth would be more strongly positively associated with life satisfaction than increases in sole wealth—own or partner’s. This was based on the idea that particularly within the context of Germany, jointness within couples is strongly encouraged and emphasised through social norms and institutional incentives. To examine whether joint wealth matters more than sole wealth for life satisfaction, we test the difference between the estimated effects of joint wealth and sole wealth once for respondents’ sole wealth and once for respondents’ partners’ sole wealth. Confirming the effect depicted in the upper panel of Fig. 2, the lower panel shows that joint wealth is more strongly positively related to subjective well-being than both sole wealth measures. The differences in the effect sizes are small but statistically significant at the 5 percent level. The differences of the effects for a one-unit increase in log-transformed joint gross wealth compared to one-unit increases in log-transformed sole or partner’s sole wealth amount to 0.010 and 0.012 life satisfaction points, respectively.

Furthermore, we anticipated in our *Sole Wealth Hypothesis* (H3) that increases in own sole wealth are more strongly positively associated with life satisfaction than increases in partner’s sole wealth. This may be because own sole wealth provides personal financial security and bargaining power, while increases in partner’s sole wealth may signal mistrust or a loss of own bargaining power. As already mentioned, the coefficient for respondents’ own sole wealth is marginally larger than the coefficient for respondents’ partner’s sole wealth. Note that both coefficients are not statistically significant at conventional levels. As shown in the lower panel of Fig. 2, the marginal difference between the coefficients is statistically not significant. Thus, we do not find support for our third hypothesis on the differences between respondents’ and their partner’s sole wealth.

**Fig. 3:**
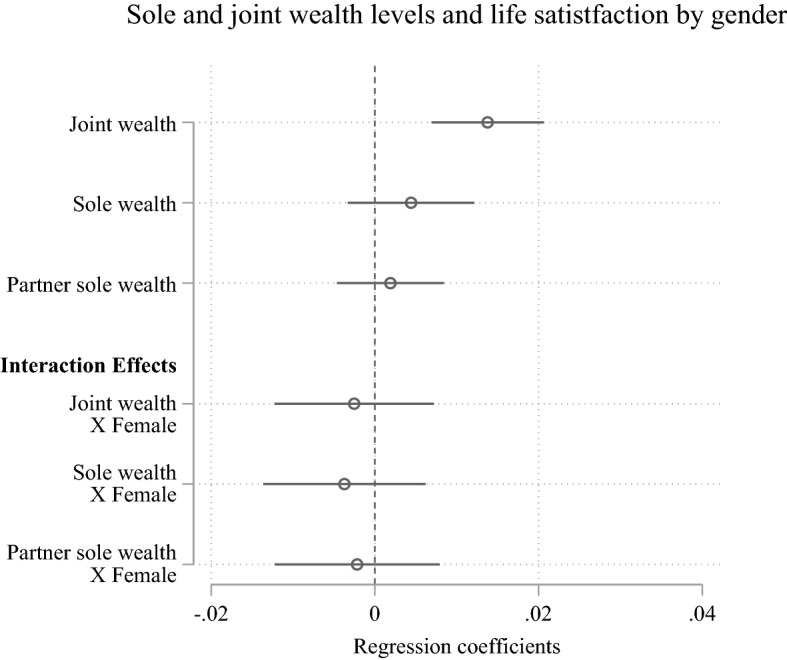
Multivariable fixed-effects regression models of sole and joint gross wealth (log-transformed) on life satisfaction including gender interaction *Notes:* Whiskers indicate 95% confidence intervals. The models are also adjusted for both partners’ personal liabilities, age, marital duration, employment level, self-employment, and income. Additionally, we account for the household’s region with regard to rural compared to urban and Western compared to Eastern Germany, and the receipt of inheritances. Full model results in Online Appendix Tables A4. *Data: SOEP (v36); multiply imputed*

Next, we argued in our *Family Provider Hypothesis* (H4) that changes in joint wealth are more strongly positively associated with life satisfaction for men than for women. This would be in line with traditional gender roles according to which men are expected to provide jointly owned assets and fulfil their role of providing financial security for the family. To test this hypothesis, we interacted the joint ownership wealth measures with gender and test if the effect of joint wealth is significantly different for women and men. Figure 3 shows that the interaction effects of joint wealth and gender are not statistically significant. Thus, our results do not support our expectation that joint wealth is more relevant for men than women. Overall, wealth changes are generally associated with lower life satisfaction improvements for women than men, although the gender differences are not statistically significant.

### Supplementary Analyses

We conduct a set of supplementary analyses to better understand the advantage of joint wealth compared to sole wealth for life satisfaction. First, we explore potential cohort differences because previous research has emphasised that recent cohorts have become more individualised in general and concerning their wealth ownership structure (Cherlin, 2004; Frémeaux & Leturcq, 2020). Further, Lersch (2017) showed that—at least for women—the level of their personal wealth (i.e. the sum of their sole and share of joint wealth) has become more strongly associated with their own financial well-being in recent cohorts. Thus, we expect that joint wealth is particularly relevant in older compared to younger cohorts and, in return, that respondents’ sole wealth is more relevant in younger than older cohorts. To assess this with our data, we conduct separate fixed-effects regression models by birth cohorts (born before or in 1945, 1946 to 1955, 1956 to 1965, and born in or after 1966). Although we find no statistically significant differences between the cohorts, the relevance of joint wealth compared to the cohorts of 1946 to 1955 and 1956 to 1965 seemed to be lower for the most recent cohorts. We find no substantial trends in sole wealth over the cohorts (see Figure A3 in Online Appendix).

Second, we disaggregate our wealth measures into financial gross and housing gross wealth. Isolating housing wealth is especially important because purchasing real estate is often a joint investment and, hence, housing wealth is most likely to be jointly owned. In contrast, financial wealth is more likely solely owned (Joseph & Rowlingson, 2012). In our analytical sample, 61 percent of respondents are homeowners. Of those, 91 percent own their property jointly with their partner (see Table 1). Supplementary results for housing and financial gross wealth reflect our main results: increases in both housing and financial joint gross wealth are associated with substantial and statistically significant life satisfaction increases (see Figure A4 and A5 in Online Appendix). Coefficients for respondents’ own or partners’ solely held financial or gross housing wealth are positive but statistically insignificant. Overall, increases in housing gross wealth are, however, associated with slightly larger increases in life satisfaction for all three wealth measures (i.e. sole, partner’s sole, joint). This may highlight the high value in terms of housing security or relationship commitment that is attributed to housing wealth.

Finally, we assess potential issues around selection out of marriage and, more broadly, attrition. Previous research showed that financial hardship is linked to a higher likelihood of divorce (Dew, 2011; Eads & Tach, 2016). At the same time, life satisfaction has also been found to be a significant predictor of marital dissolution (van Scheppingen & Leopold, 2020). In total, 350 female anchor respondents experience the dissolution of their marriage during the observational window, while this is the case for only 203 male anchor respondents. Note that the difference is due to women’s higher likelihood to experience widowhood and potentially gendered attrition after separation and divorce. Descriptive differences between the two groups reveal that respondents that eventually experience a marital dissolution have marginally lower life satisfaction and overall less wealth, but also lower debt levels compared to continuously married respondents (see Table A5 in Online Appendix). Using a regression framework (see Table A6 in Online Appendix), we find that life satisfaction and our three gross wealth measures predict both the likelihood to experience a marital dissolution during the observational window and attrition. However, the coefficients are relatively small compared to those for age or employment. Further analyses also show that the marital dissolution dummy and the attrition dummy do not directly predict life satisfaction or moderate the association between wealth and life satisfaction. Thus, in sum, we do not find strong evidence that should raise concerns about selection.

## Discussion

This study examines the money-subjective well-being nexus by considering the link between, on the one hand, changes in jointly and solely held gross wealth and, on the other hand, changes in married individuals’ subjective well-being. We argued that how spouses accumulate wealth within marriage may contribute to their subjective well-being. The link between how wealth ownership is distributed between spouses and their subjective well-being is central in light of recent trends of financial individualisation in marriage against the background of the Second Demographic Transition (Van De Kaa, 1987). We expected increases in solely held wealth, partner’s solely held wealth, and jointly held wealth to positively affect well-being (*H1 Wealth hypothesis*). However, we also anticipated differences in the degree to which increases in well-being are dependent on how wealth is held. Increases in joint assets were expected to be more strongly positively related to subjective well-being than solely held wealth because they reflect cultural norms of sharing responsibilities and resources (*H2 Joint wealth hypothesis*). Furthermore, increases in solely held assets were expected to be stronger positively associated with well-being when compared to partner’s solely held wealth, because increases in own solely held wealth support individual economic independence and security (*H3 Sole wealth hypothesis*). In contrast, increases in partners’ solely held wealth may be perceived as a sign of increasing mistrust in the marriage. Because of gendered expectations regarding the financial provider role, men’s well-being may depend more strongly on increases in joint wealth than women’s well-being (*Hypothesis 4 Family provider hypothesis*).

We study the country case of Germany. The German tax system and welfare provisions incentivise joint investments and promote a gender-traditional division of labour (Bach et al., 2013; Buslei & Wrohlich, 2014). At the same time, individualisation tendencies are also clearly visible in Germany, with substantial within-couple wealth gaps (Kapelle & Lersch, 2020). Overall, this makes Germany a particularly interesting case for the current study. Using comprehensive longitudinal data on personal wealth ownership from the SOEP, we examined whether changes in solely and jointly held wealth are related to changes in spouses’ life satisfaction.

Overall, while effect sizes for all three wealth measures are positive, we find substantial differences in the association between the three types of wealth ownership and life satisfaction. First, only increases in joint wealth are statistically positively related to subjective well-being. The positive coefficients for the two sole wealth measures are not statistically significant at conventional levels and substantially below effect sizes for joint wealth. Furthermore, we find no indication of substantially different effects between own sole wealth and partner’s sole wealth on life satisfaction within marriage. Thus, increases in jointly held wealth are most relevant for improving spouses’ subjective well-being while increases in either partner’s solely held wealth are less relevant, at least within the rather traditional context of Germany.

Furthermore, we did not find substantial gender differences in the positive association between increases in joint wealth and life satisfaction. Men do not seem to gain more life satisfaction from their role as active financial providers for the family compared with their female partners. This indicates that fulfilling norms of marital jointness by accumulating wealth jointly is beneficial for both partners: In Germany, having joint wealth seems to be an indicator of higher conformity to the cultural script of sharing, which is linked to higher life satisfaction compared with owning sole wealth. The fact that Germany is characterised by several institutional incentives to think and act jointly does add plausibility to this association.

We further show that how specific wealth components are held within couples seems to matter differently for individuals’ life satisfaction. This is the case for housing wealth, which is most couples’ most significant joint investment that might send a strong signal of marital commitment that cannot be expressed with other joint financial assets. Thus, our results indicate that the wealth ownership structure within couples shapes individuals’ life satisfaction in interplay with other characteristics of the wealth that partners hold in their portfolios.

These results make at least two contributions to the literature on the relationships between marriage, wealth, and subjective well-being. First, we show that the well-established positive association between wealth and life satisfaction is not exclusively driven by own need fulfilment and social comparisons but also seems to depend on social norms (Diener & Biswas-Diener, 2002). Our study indicates that resource sharing in line with the marital script considerably increases individuals’ life satisfaction within marriage. Also, in light of the individualisation of marriages (Yodanis & Lauer, 2014), the personal benefits associated with marital sharing seem to trump those of economic independence and financial autonomy.

Second, we enrich the literature on the money-subjective well-being nexus both theoretically and empirically by adopting a within-couple perspective on the study of wealth and subjective well-being. While a handful of studies have already contributed to this area of research (Kan & Laurie, 2014; Lersch, 2017; Tisch, 2021), we disaggregate the types of wealth ownership within couples fully and show that how wealth is held matters for subjective well-being beyond couple’s total wealth or personal wealth (measured as solely held plus share of jointly held wealth). Our results highlight that joint wealth seems to play the most important role. This could be partly driven by the fact that money is loaded with social meaning (Zelizer, 1989), implying different *monies*—depending on the property rights and social relationships within the household.

This work is not without limitations, which highlight potential avenues for future data collection and research. First, we are unable to rely on tax records or other register data to directly observe the property rights of spouses. Instead, survey data reflect subjective reports of wealth holdings, which may be inaccurate, for instance, because respondents misconceive the matrimonial property regime (Joseph & Rowlingson, 2012). However, the SOEP data are unique in measuring wealth longitudinally and at the individual level, even within households. Second, we limit our analyses to married couples, as long-term cohabitation is still residual for the birth cohorts in our sample. Long-term cohabitation is increasing among younger cohorts, so future research may extend to cohabiting couples once data are available. This future research avenue of considering cohabiting couples is critical because increasing cohabitation rates may not only shape the prevailing cultural scripts, but cohabiting couples also rely on specific legal rights and obligations attached to it. See Vitali and Fraboni (2022) in this Special Issue for an analysis of wealth management differences between cohabiting and married couples in Italy including a focus on pre-marital cohabitation. The question of whether the relationship between personal property rights and well-being differs between cohabiting and married couples remains open: For cohabiting couples, sole ownership might play a more critical role in predicting well-being even in Germany because there are no legal protections for the case of union dissolution after cohabitation. However, for long-term cohabitors, associations between property rights and well-being might become more similar to those in marriage.

Notwithstanding these limitations, the results discussed above represent a pivotal steppingstone towards a better understanding of how the way different wealth components are held within couples is related to life satisfaction. Our results have, thus, important implications for research in demography, sociology, and economics looking at the link between financial arrangements and well-being, which is also relevant for outcomes such as health, longevity, and parenthood.

While our research could not examine the link between the trends in individualisation of marriage and preferences for keeping money separate, our results indicate that this process may be further along due to institutional structures favouring individual savings and investments. Our results suggest that marriage is a heterogeneous social institution that needs to be carefully unpacked to understand the underlying social and economic relationships. Although there seems to be a trend towards individualisation in marriage, accumulating wealth jointly remains essential for individual’s well-being.

Finally, this study adds relevant evidence to the critical discussion of unitary versus individualised trends within marriage in the field of economic inequality. Recently, a growing body of wealth research as argued against a unitary household model (e.g. Lersch, 2017; Tisch, 2021). This is not to say that a fully individualised account without consideration of household-level dependencies and resource sharing would be adequate. Instead, sharing and separateness of resources coexist and need to be jointly accounted for. In the study of wealth inequality, however, the household is still often the exclusive research unit of choice, assuming full sharing. Our results strongly suggest that this choice ignores relevant within-household differences in resources even when considering married people. Although we find that increases in jointly held wealth rather than solely held wealth are most relevant for life satisfaction—likely due to the relevance of joint wealth for homeownership, our results show that the share of solely held wealth in couples is non-negligible and solely held wealth likely has a range of advantages beyond jointly held wealth. For instance, solely held wealth may be particularly relevant for individuals’ ability to leave an unsatisfying relationship. Thus, research on the link between wealth and divorce would be well-advised to consider the ownership structure of wealth within couples (see Eads and Tach (2016) for a first step in this direction).

## Supplementary Information

Below is the link to the electronic supplementary material.

Supplementary file1 (PDF 623 kb)

## Footnotes

1 Sample respondents are on average 56 years of age, but age ranges from 19 to 97 years.

2 See Rehm, Schneebaum, and Schuster (2022) in this Special Issue for an overview of relevant demographic dimensions that importantly determine within-couple wealth differences.

3 Unfortunately, we are unable to account for gender ideology. The causes and consequences of how wealth is divided within couples likely differ substantially by spouses normative understanding of such processes. Particularly for gender differences it would be intriguing for future research to explore the heterogeneity in wealth ownership and subjective well-being by individuals’ gender ideologies.
