# Supplementary material for: My Wealth, (Y)Our Life Satisfaction? Sole and Joint Wealth Ownership and Life Satisfaction in Marriage
Source: Eur J Popul. 2022 Aug 30;38(4):811–34. doi: 10.1007/s10680-022-09630-7 (PMC9550900; doi:10.1007/s10680-022-09630-7)
Supplement: Supplementary file 1 — Supplementary file1 (PDF 623 kb) [file 10680_2022_9630_MOESM1_ESM.pdf]

Online appendix

My wealth, (y)our life satisfaction? Sole and joint wealth ownership and life satisfaction in marriage

Supplementary Tables

Table A1 Description of wealth measurement in SOEP

**Type of wealth**	**Wealth components**
Financial wealth	Savings account / bonds / shares / investments (*Personal share collected if any of these co-owned*)
	Building loan agreement / life insurance policy / private retirement plan
	Tangible assets (gold / jewelry / coins / valuable collections)
Housing wealth	Primary housing market value (*Personal share collected if co-owned*)
	Remaining mortgage (*Personal share collected if co-owned*)
	Other real estate market value (*Personal share collected if co-owned*)
	Remaining mortgage (*Personal share collected if co-owned*)
Business assets	Company / shop / office / practice / farm (*Personal share collected if held jointly*)
Vehicles	Car / motorcycle / motor home / privately used truck (only measured in 2017) *
Debts	Owe money on loans (bank / other institution / other person)
	Student loan (only measured in 2017)*

*Wealth component not considered for current manuscript to ensure consistency over time.

Note: For further information on the SOEP wealth component see Grabka and Westermeier (2015).

Table A2 Overview of variables used for the imputation models including number and shares of missing values in Germany

		2002		2007		2012		2017	
		Nr. of missing values	Percentage	Nr. of missing values	Percentage	Nr. of missing values	Percentage	Nr. of missing values	Percentage
Outcome variable	Life satisfaction	10	0.13	18	0.21	22	0.25	8	0.12
Wealth variables*	Financial joint wealth+				none				
	Financial sole wealth+				none				
	Financial partner's sole wealth+				none				
	Housing joint wealth+				none				
	Housing sole wealth+				none				
	Housing partner's sole wealth+				none				
	Debt+				none				
	Partner's debt+				none				
Variables used for main analyses	Age				none				
	Partner's age				none				
	marital duration	17	0.23	16	0.19	3	0.03	0	0.00
	Urban				none				
	Federal states§				none				
	Inheritance	100	1.33	262	3.06	239	2.76	368	5.55
	Employment level				none				
	Partner's employment level				none				
	Self-employed				none				
	Partner self-employed				none				
	Personal income+				none				
	Partner's personal income+				none				
	Gender				none				
Other auxiliary variables	SOEP Sample				none				
	Migration background				none				
	Partner's migration background				none				
	Cohort				none				
	Financial preparedness	410	5.44	518	6.05	627	7.25	304	4.58
	Partner's financial preparedness	410	5.44	518	6.05	627	7.25	304	4.58

Financial concerns	20	0.27	30	0.35	28	0.32	24	0.36
Partner's financial concerns	20	0.27	30	0.35	28	0.32	24	0.36
Employment status					none			
Partner's employment status					none			
HH net income+					none			
Children 0 to 4					none			
Children 5 to 10					none			
Children 11 to 18					none			
Nr of children ever had					none			
Partner's nr of children ever had					none			
Education	11	0.15	15	0.18	4	0.05	3	0.05
Partner's education	11	0.15	15	0.18	4	0.05	3	0.05
Father's education	923	12.25	1006	11.76	718	8.31	477	7.19
Partner's father's education	923	12.25	1006	11.76	718	8.31	477	7.19
Mother's education	929	12.33	1008	11.78	724	8.38	463	6.98
Partner's mother's education	929	12.33	1008	11.78	724	8.38	463	6.98
Health	9	0.12	21	0.25	8	0.09	9	0.14
Partner's health	9	0.12	21	0.25	8	0.09	9	0.14
Partner's life satisfaction	10	0.13	18	0.21	22	0.25	8	0.12

Notes: *We impute financial gross and housing gross wealth measures instead of total gross wealth. This is due to the fact that the sum of financial and housing wealth equates to the total of gross wealth. Thus, after the imputation we sum financial and housing wealth to generate the total gross wealth measures we use in our main models. §Federal states are aggregated after the imputation to generate a dummy that indicates whether a couple is living in Eastern or Western Germany. + In our imputation models for the SOEP data, we include income and personal wealth measures that were edited and imputed by the SOEP team. The SOEP team imputed income data for under 10 percent of respondents (Frick & Grabka, 2014). As personal wealth includes a range of measures, the amount of missing values differs between the different wealth components. Overall, incidences of item-nonresponse are however rather low and vary between zero percent for debts measures on other property (i.e. property other than the primary home) to about 14 percent for information on private insurance (Grabka & Westermeier, 2015).

Table A3 Descriptive statistics.

	Total				Women				Men			
	Mean/ Prop	SD	Min	Max	Mean/ Prop	SD	Min	Max	Mean/ Prop	SD	Min	Max
Outcome measure:												
Life satisfaction	8.25	1.61	1.00	11.00	8.28	1.61	1.00	11.00	8.22	1.61	1.00	11.00
Wealth levels and portfolio:												
Sole gross wealth (EUR)	52.97	276.99	0.00	23720.00	30.73	115.98	0.00	4070.60	75.21	372.84	0.00	23720.00
Sole gross wealth partner (EUR)	53.12	279.84	0.00	23720.00	75.56	376.37	0.00	23720.00	30.67	118.18	0.00	4331.30
Joint gross wealth	93.86	164.67	0.00	7520.00	92.83	155.92	0.00	3328.00	94.90	172.98	0.00	7520.00
Personal debts and liabilities	23.72	67.67	0.00	4250.00	21.16	49.28	0.00	1400.00	26.28	81.95	0.00	4250.00
Partner debts and liabilities	23.78	68.00	0.00	4250.00	25.76	69.75	0.00	3255.00	21.79	66.14	0.00	4250.00
Homeownership (ref. no homeownership)	0.61		0.00	1.00	0.61		0.00	1.00	0.61		0.00	1.00
Sole homeownership (ref. joint)	0.09		0.00	1.00	0.06		0.00	1.00	0.12		0.00	1.00
Other covariates												
Age	53.18	14.13	19.00	97.00	51.87	14.05	19.00	92.00	54.49	14.08	22.00	97.00
Partner’s age	53.18	14.13	19.00	97.00	54.49	14.08	22.00	97.00	51.87	14.05	19.00	92.00
Cohort												
-1945	0.27		0.00	1.00	0.24		0.00	1.00	0.30		0.00	1.00
1946-1955	0.19		0.00	1.00	0.20		0.00	1.00	0.19		0.00	1.00
1956-1965	0.23		0.00	1.00	0.23		0.00	1.00	0.24		0.00	1.00
1966-	0.30		0.00	1.00	0.33		0.00	1.00	0.27		0.00	1.00
Marital duration	28.23	15.56	1.00	73.00	28.23	15.56	1.00	73.00	28.22	15.56	1.00	71.00
Nr of children in household												
Aged 0 to 4	0.15	0.42	0.00	4.00	0.15	0.42	0.00	4.00	0.15	0.42	0.00	4.00
Aged 5 to 10	0.30	0.63	0.00	5.00	0.30	0.63	0.00	5.00	0.30	0.63	0.00	5.00

Aged 11 to 18	0.37	0.72	0.00	5.00	0.37	0.72	0.00	5.00	0.37	0.72	0.00	5.00
Nr of children ever had	1.94	1.15	0.00	12.00	2.09	1.09	0.00	12.00	1.80	1.19	0.00	12.00
Partner's nr of children ever had	1.94	1.15	0.00	12.00	1.80	1.19	0.00	12.00	2.09	1.09	0.00	12.00
Education												
Low	0.09		0.00	1.00	0.12		0.00	1.00	0.06		0.00	1.00
Intermediate	0.58		0.00	1.00	0.61		0.00	1.00	0.55		0.00	1.00
High	0.34		0.00	1.00	0.27		0.00	1.00	0.40		0.00	1.00
Father's education												
Low	0.37		0.00	1.00	0.37		0.00	1.00	0.38		0.00	1.00
Intermediate	0.49		0.00	1.00	0.49		0.00	1.00	0.48		0.00	1.00
High	0.14		0.00	1.00	0.14		0.00	1.00	0.14		0.00	1.00
Mother's education												
Low	0.60		0.00	1.00	0.58		0.00	1.00	0.61		0.00	1.00
Intermediate	0.35		0.00	1.00	0.36		0.00	1.00	0.34		0.00	1.00
High	0.05		0.00	1.00	0.06		0.00	1.00	0.05		0.00	1.00
Employment level												
Full-time	0.40		0.00	1.00	0.18		0.00	1.00	0.61		0.00	1.00
Part-time/irregular	0.20		0.00	1.00	0.36		0.00	1.00	0.04		0.00	1.00
Not employed/other	0.40		0.00	1.00	0.46		0.00	1.00	0.34		0.00	1.00
Self-employed (ref. not self-employed	0.07		0.00	1.00	0.05		0.00	1.00	0.09		0.00	1.00
Annual labour income in EUR	22.49	32.59	0.00	1280.00	12.08	16.86	0.00	240.00	32.90	40.29	0.00	1280.00
Born abroad (ref. not born abroad)	0.13		0.00	1.00	0.13		0.00	1.00	0.13		0.00	1.00
Urban (ref. rural)	0.65		0.00	1.00	0.65		0.00	1.00	0.65		0.00	1.00
Eastern Germany (ref. Western)	0.24		0.00	1.00	0.24		0.00	1.00	0.24		0.00	1.00
Obs.: Couples/ ind.		11,732				5,866				5,866		
Couples/individuals		31,370				15,685				15,685		

Notes: Wealth, debts, and annual income reported in thousand EUR. Data are from the Socio-Economic Panel Survey (v36; unweighted; multiply imputed).

Table A4 Fixed-effects regression models of sole and joint gross wealth (log-transformed) on life satisfaction.

	Base model<br><br>Life satisfaction (11 scale)<br>b/se/p	Incl. gender interaction<br>Life satisfaction (11 scale)<br>b/se/p
Joint gross wealth (log-transformed)	0.012	0.014
	(0.003)	(0.003)
	[0.000]	[0.000]
Sole gross wealth (log-transformed)	0.002	0.004
	(0.003)	(0.004)
	[0.352]	[0.258]
Sole gross wealth partner (log-transformed)	0.001	0.002
	(0.003)	(0.003)
	[0.723]	[0.566]
Debt (log-transformed)	-0.004	-0.004
	(0.004)	(0.004)
	[0.314]	[0.321]
Partner debt (log-transformed)	-0.002	-0.002
	(0.004)	(0.004)
	[0.505]	[0.493]
Age brackets (ref. up to 39)		
40 to 49	-0.080	-0.079
	(0.037)	(0.037)
	[0.032]	[0.033]
50 to 59	-0.198	-0.198
	(0.057)	(0.057)
	[0.000]	[0.000]
60 to 69	-0.121	-0.122
	(0.074)	(0.074)
	[0.102]	[0.098]
70 and older	-0.191	-0.192
	(0.088)	(0.088)
	[0.031]	[0.030]
Partner’s age brackets (ref. up to 39)		
40 to 49	-0.114	-0.115
	(0.037)	(0.037)
	[0.002]	[0.002]
50 to 59	-0.190	-0.190
	(0.057)	(0.057)
	[0.001]	[0.001]
60 to 69	-0.140	-0.139
	(0.073)	(0.073)
	[0.056]	[0.058]
70 and older	-0.272	-0.271
	(0.088)	(0.088)
	[0.002]	[0.002]
Marital duration (ref. up to 9 years)		
10 to 19	-0.031	-0.031
	(0.037)	(0.037)
	[0.402]	[0.405]
20 to 29	-0.061	-0.061
	(0.058)	(0.058)
	[0.287]	[0.288]

30 and more	0.006	0.006
	(0.076)	(0.076)
	[0.942]	[0.941]
Region type urban (ref. rural)	0.041	0.040
	(0.105)	(0.105)
	[0.698]	[0.703]
Living in East Germany (ref. West Germany)	-0.384	-0.384
	(0.185)	(0.185)
	[0.038]	[0.038]
Inheritance received in last years (ref. no inheritance)	0.028	0.028
	(0.031)	(0.031)
	[0.368]	[0.366]
Employment level (ref. full-time)		
Part-time/irregular	-0.060	-0.058
	(0.038)	(0.038)
	[0.122]	[0.133]
Not employed/other	-0.171	-0.170
	(0.047)	(0.047)
	[0.000]	[0.000]
Partner’s employment level (ref. full-time)		
Partner part-time/irregular	-0.012	-0.014
	(0.038)	(0.038)
	[0.743]	[0.705]
Partner not employed/other	-0.061	-0.062
	(0.044)	(0.044)
	[0.166]	[0.162]
Self-employed (ref. not self-employed)	-0.005	-0.006
	(0.064)	(0.064)
	[0.933]	[0.920]
Partner self-employed (ref. not self-employed)	-0.144	-0.143
	(0.056)	(0.056)
	[0.010]	[0.010]
Annual income (log-transformed)	-0.006	-0.006
	(0.004)	(0.004)
	[0.151]	[0.148]
Partner annual income (log-transformed)	-0.001	-0.001
	(0.004)	(0.004)
	[0.877]	[0.882]
Joint gross wealth (log-transformed) X Female		-0.003
		(0.005)
		[0.611]
Sole gross wealth (log-transformed) X Female		-0.004
		(0.005)
		[0.459]
Sole gross wealth partner (log-transformed) X Female		-0.002
		(0.005)
		[0.671]
Obs.: Couples / individuals	11,732 / 5,866	
Couples / individuals	31,370 / 15,685	

Notes: Data are from the Socio-Economic Panel Survey (v36; unweighted; multiply imputed).

Table A5 Descriptive statistics comparing sample respondents that do not experience a marital dissolution during the observation window and respondents that experience a marital dissolution.

	Continuously married over observational window				Experience dissolution during observational window			
	Mean/Prop	SD	Min	Max	Mean/Prop	SD	Min	Max
Outcome measure:								
Life satisfaction	8.26	1.60	1.00	11.00	8.01	1.67	1.00	11.00
Wealth levels and portfolio:								
Sole gross wealth (EUR)	54.02	282.24	0.00	23720.00	28.73	91.64	0.00	1069.35
Sole gross wealth partner (EUR)	52.76	279.50	0.00	23720.00	61.34	287.49	0.00	5005.40
Joint gross wealth	94.84	166.20	0.00	7520.00	71.26	122.28	0.00	1349.70
Personal debts and liabilities	24.01	68.49	0.00	4250.00	16.93	44.26	0.00	450.63
Partner debts and liabilities	23.94	68.51	0.00	4250.00	19.97	54.67	0.00	564.32
Homeownership (ref. no homeownership)	0.62		0.00	1.00	0.53		0.00	1.00
Sole homeownership (ref. joint homeownership)	0.09		0.00	1.00	0.07		0.00	1.00
Observations	30,065				1,305			
Individuals	11,179				553			

Notes: Wealth, debts, and annual income reported in thousand EUR. Data are from the Socio-Economic Panel Survey (v36; unweighted; multiply imputed).

Table A6 Assessing attrition and selection using probit and OLS regression.

	Assessing attrition bias		Assess bias due selection out of marriage	
	Predicting attrition	Predicting life satisfaction	Predicting marital dissolution	Predicting life satisfaction
	B/(SE)	B/(SE)	B/(SE)	B/(SE)
Life satisfaction (11 scale)	-0.07***		-0.03**	
	(0.01)		(0.01)	
Attrition (ref. no)		-0.10		
		(0.15)		
Dissolution (ref. no dissolution)				0.53
				(0.37)
Wealth measures				
Joint gross wealth (log-transformed)	-0.01***	0.04***	-0.01*	0.04***
	(0.00)	(0.00)	(0.00)	(0.00)
Sole gross wealth (log-transformed)	-0.01***	0.02***	-0.00	0.02***
	(0.00)	(0.00)	(0.00)	(0.00)
Sole gross wealth partner (log-transformed)	-0.01***	0.01***	-0.00	0.01***
	(0.00)	(0.00)	(0.00)	(0.00)
Interactions				
Attrition X Joint gross wealth (log-transformed)		0.01		
		(0.00)		
Attrition X Sole gross wealth (log-transformed)		0.00		
		(0.01)		
Attrition X Sole gross wealth partner (log-transformed)		-0.00		
		(0.00)		
Dissolution X Joint gross wealth (log-transformed)				-0.01
				(0.01)
Dissolution X Sole gross wealth (log-transformed)				-0.00
				(0.01)
Dissolution X Sole gross wealth partner (log-transformed)				-0.01
				(0.01)
Observations	31,370			
Individuals	11,732			

Notes: The models are also adjusted for both partners' personal liabilities, age, employment level, self-employment, and income. Additionally, models are accounted for marital duration, the household' receipt of inheritances in the last years compared to not receiving any inheritances, the household's region with regard to rural compared to urban and Western compared to Eastern Germany. Data are from the Socio-Economic Panel Survey (v36; unweighted; multiply imputed).

Supplementary Figures

Figure A1 Distribution of subjective well-being

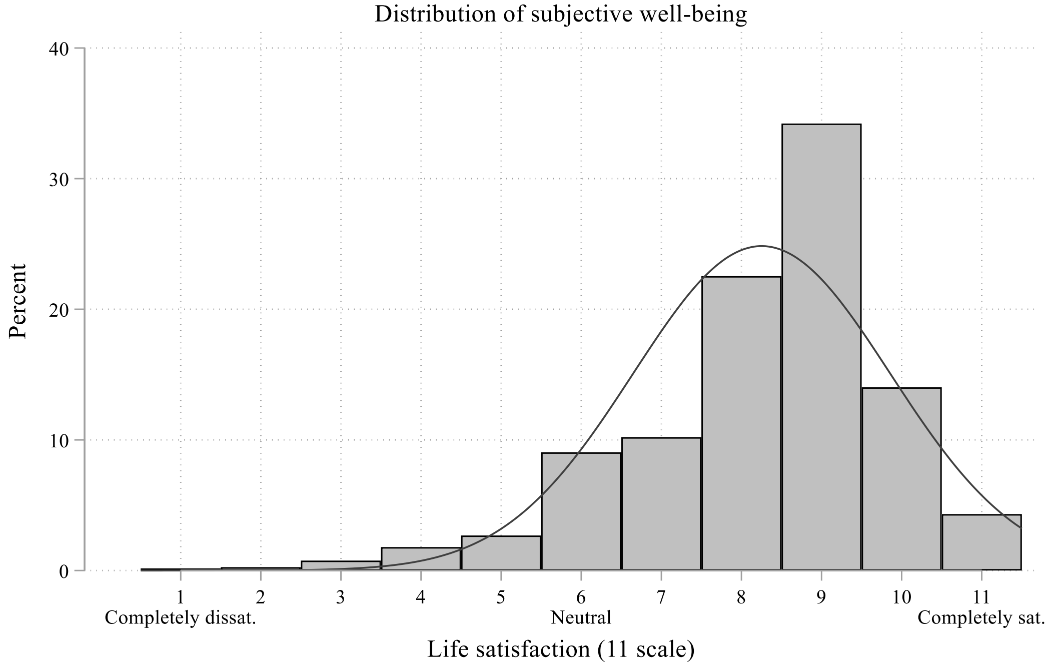

Notes: Data are from the Socio-Economic Panel Survey (v36; unweighted; multiply imputed).

Figure A2 Multivariable fixed-effects regression models of sole and joint gross wealth (log-transformed) on standardised life satisfaction adjusted for the presence, number and age of dependent children ages 0 to 18 living in the household, and respondents' and their partner's number of children ever had

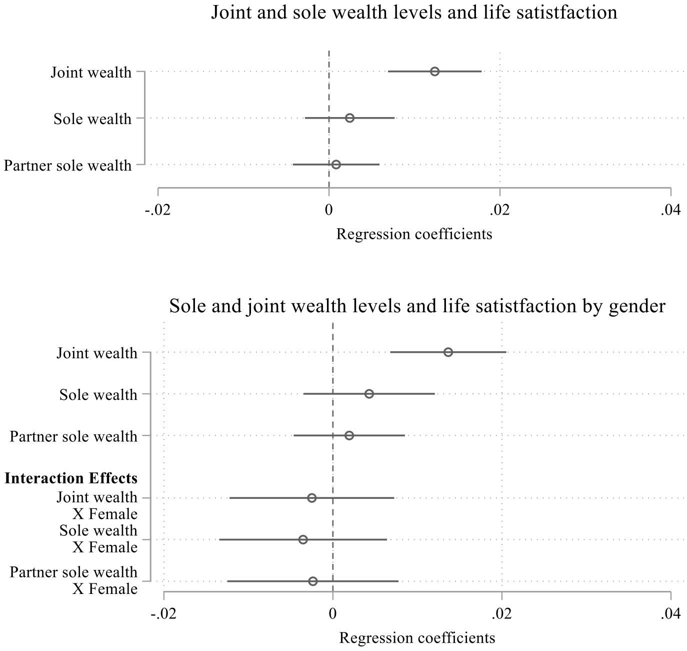

Notes: Whiskers indicate 95% confidence intervals. Data are from the Socio-Economic Panel Survey (v36; unweighted; multiply imputed).

Figure A3 Multivariable fixed-effects regression models of sole and joint gross wealth (log-transformed) on standardised life satisfaction by cohorts

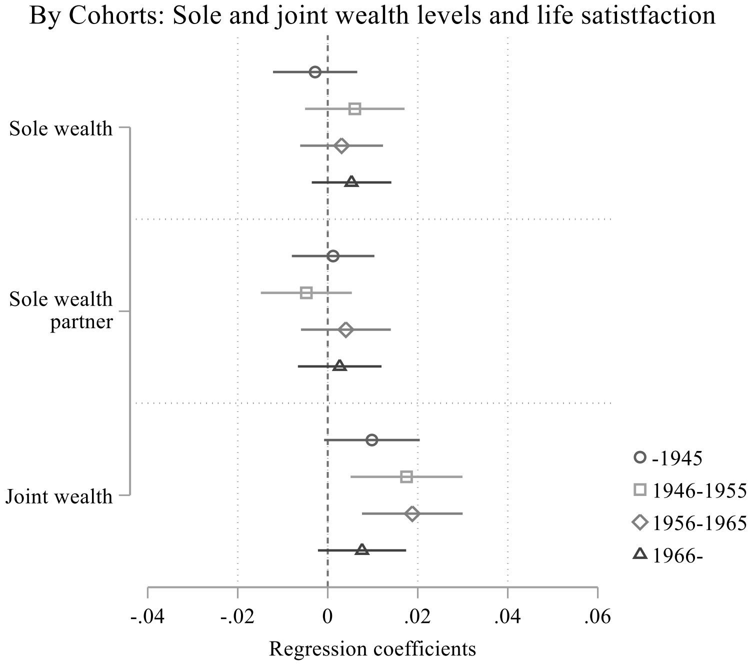

Notes: Whiskers indicate 95% confidence intervals. Data are from the Socio-Economic Panel Survey (v36; unweighted; multiply imputed).

Figure A4 Multivariable fixed-effects regression models of sole and joint gross financial wealth (log-transformed) on standardised life satisfaction

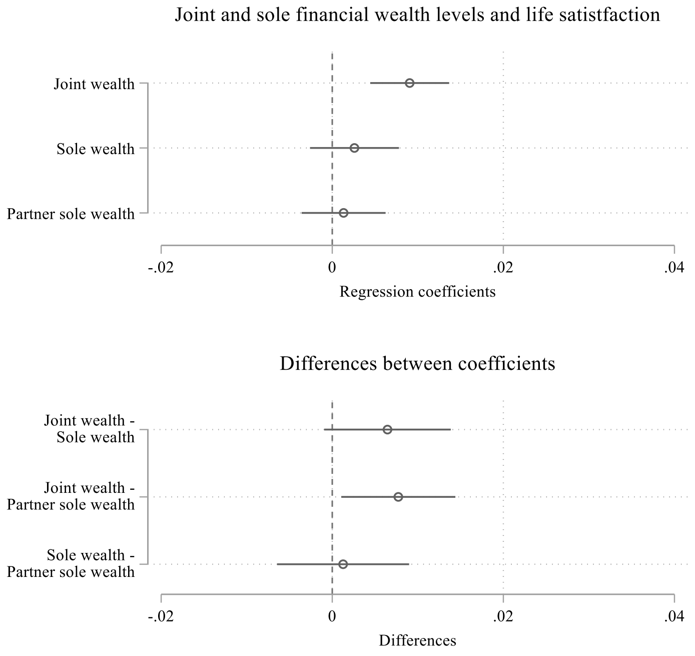

Notes: Whiskers indicate 95% confidence intervals. Data are from the Socio-Economic Panel Survey (v36; unweighted; multiply imputed).

Figure A5 Multivariable fixed-effects regression models of sole and joint gross housing wealth (log-transformed) on standardised life satisfaction

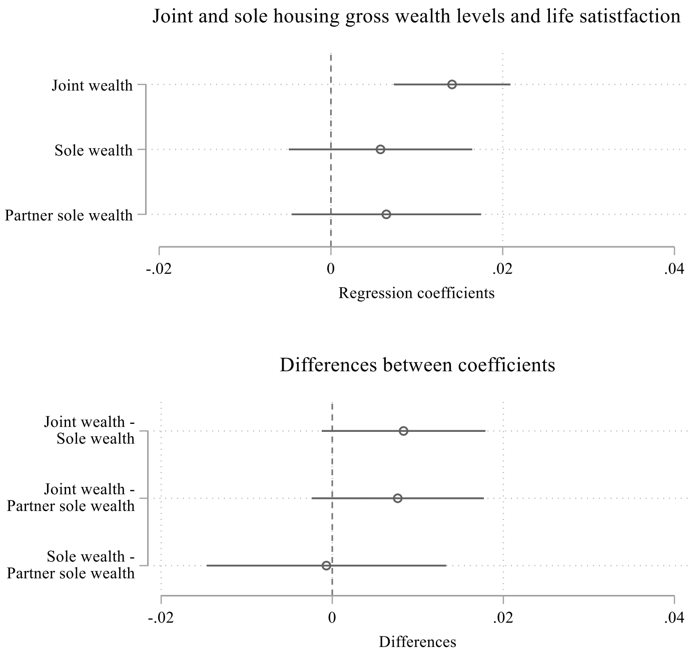

Notes: Whiskers indicate 95% confidence intervals. Data are from the Socio-Economic Panel Survey (v36; unweighted; multiply imputed).

References

Frick, J. R., & Grabka, M. M. (2014). *Missing income data in the German SOEP: Incidence, imputation and its impact on the income distribution*. Retrieved from Berlin, Germany:

Grabka, M. M., & Westermeier, C. (2015). *Editing and multiple imputation of item non-response in the wealth module of the German Socio-Economic Panel*. SOEP Survey Papers, Series C - Data Documentation, No. 272. DIW, Berlin, Germany.
